# High-frequency oscillation improves mucus clearance and airway resistance in mechanically ventilated patients: a randomized clinical trial

**DOI:** 10.1016/j.aicoj.2026.100062

**Published:** 2026-04-09

**Authors:** Yuxuan Wang, Dongyu Chen, Wei Xie, Rui Zhang, Xinxin Guo, Haiying Wu, Ling Liu, Xueyan Yuan

**Affiliations:** a Department of Emergency, Central Hospital Affiliated to Shandong First Medical University, Shandong First Medical University & Shandong Academy of Medical Sciences, Jinan, China; b Jiangsu Provincial Key Laboratory of Critical Care Medicine, Department of Critical Care Medicine, Zhongda Hospital, School of Medicine, Southeast University, Nanjing, 210009, China; c Department of Critical Care Medicine, The First people’s Hospital of Yancheng, Yancheng No.1 People’s Hospital, Affiliated Hospital of Medical School, Nanjing University, Yancheng, 224000, China; d Department of Anesthesiology and Surgery, Shandong Rehabilitation Research Center, Shandong Rehabilitation Hospital, Jinan, China; e Emergency Department, First Affiliated Hospital of Kunming Medical University, Kunming 650500, China

**Keywords:** Keyword Continuous high-frequency oscillation, excessive airway secretions, airway resistance, Respiratory system compliance

## Abstract

**Background:**

Mechanical ventilation is associated with both impaired ciliary function and a weakened cough, which further impair secretion clearance. Continuous high-frequency oscillation (CHFO) is a promising technique to reduce respiratory muscle loading and facilitate secretion clearance in mechanically ventilated patients. The aim of this study was to assess the effect of CHFO on airway resistance (Raw).

**Methods:**

This is a prospective, randomized controlled trial conducted in a 60-bed ICU between April 2023 and March 2024. Mechanically ventilated patients with excessive airway secretions (defined as the sputum volume exceeding 150 ml within the past 24 hours) were randomly assigned to either receive CHFO for 10 minutes followed by secretion aspiration (CHFO group), or to undergo secretion aspiration alone (control group). Throughout the study, only the study intervention and necessary suctioning were performed, and patient positioning and ventilator settings were kept constant. Arterial blood gases, respiratory mechanics, and the percentage of dorsal lung ventilation (assessed by electrical impedance tomography) were measured in both groups at four timepoints: pre-intervention (baseline), immediately post-intervention (T0), one-hour post-intervention (T1), and three-hours post-intervention (T3). The primary outcome was the change in Raw from baseline at each time point (ΔRaw: R_post-intervention_ - R_baseline_). Change in respiratory system compliance (Crs), and ventilation distribution were also recorded.

**Results:**

46 patients, with a median sputum volume of 160 ml over the last 24 hours, were enrolled. Baseline characteristics were well-balanced in the two groups. CHFO group showed a significantly larger decrease in Raw compared to control group at T1 (CHFO: -2.4 ± 1.8 vs. control: -0.1 ± 1.6 cmH₂O/L·s, p < 0.001) and T3 (CHFO: -1.8 ± 2.4 vs. control: -0.5 ± 1.9 cmH₂O/L·s, p < 0.05). Increase in Crs from baseline was greater in the CHFO group than control group at T1 (CHFO: 4.9 ± 8.8 vs. control: 0.3 ± 4.2 ml/cmH_2_O, p < 0.05) and T3 (CHFO: 4.3 ± 9.6 vs. control: 1.0 ± 5.8 ml/cmH_2_O, p < 0.05). Increase in dorsal lung ventilation from baseline was greater in the CHFO group compared to control group to T1 (p < 0.05). No differences in oxygenation were observed between the two groups at any time point.

**Conclusions:**

In mechanically ventilated patients with excessive airway secretions, the reduction in Raw from baseline was significantly greater in the CHFO group than control group at one-hour and three-hour post-intervention.

## Background

Mechanical ventilation (MV) is a common form of respiratory support in intensive care units (ICUs). Despite its widespread use, MV is associated with both impaired ciliary function and a weakened cough, which further impair secretion clearance [[1], [2], [3], [4]]. Inadequate clearance of airway secretions can lead to various complications, including atelectasis, mucus plugging, and recurrent pneumonia [[5], [6], [7]]. Evidence suggests that impaired mucus clearance in critically ill patients is associated with poorer clinical outcomes [8,9]. Airway clearance techniques (ACTs) have been demonstrated to optimize patients’ clinical outcomes and expedite recovery following acute illnesses [4,[10], [11], [12]]. Although the benefits of ACTs are well-established, these methods remain relatively ineffective in treating atelectasis and often fail to clear peripheral secretions [13,14].

Continuous high-frequency oscillation (CHFO) is an airway clearance technique designed to generate a global effect of internal lung percussion, aimed at clearing peripheral bronchial secretions [[15], [16], [17]]. CHFO has been shown to improve secretion clearance by furnishing a convective front of gas to the distal airways. Evidence supports the clinical effectiveness of CHFO in patients with chronic obstructive pulmonary disease exacerbations and in those receiving mechanical ventilation [[18], [19], [20], [21]]. Recently, our multicenter randomized controlled trial also confirmed that CHFO significantly reduced nonaerated lung tissue (assessed by CT scan) in mechanically ventilated patients with impaired consciousness [22]. However, the physiological effect of CHFO remains unclear when it is specifically used as a strategy to facilitate secretion removal in mechanically ventilated patients.

The primary aim of this study was to assess the physiological effect of CHFO on respiratory mechanics and lung ventilation distribution in mechanically ventilated patients. We hypothesized that CHFO could decrease the airway resistance (Raw), improve respiratory system compliance (Crs), and homogenize the ventilation distribution compared to usual care.

## Methods

### Study design

This was a single-center, randomized controlled trial conducted in a 60-bed ICU at Zhongda Hospital, Southeast University China. The study was approved by the Institutional Review Board of Zhongda Hospital (NO. 2023ZDSYLL108-P01) and registered in Chinese Clinical Trial Registry (No. ChiCTR2300070988; available at https://www.chictr.org.cn; date of registration: April 27, 2023). Informed consent was obtained according to the local regulations. The present report follows the Consolidated standards of Reporting Trials (CONSORT) reporting guideline [23].

### Patients

Consecutive patients who were admitted to our ICU were screened. Mechanically ventilated patients were eligible if they were between 18 and 80 years old and had excessive airway secretions (more than 150 mL within a 24 -h period). Patients were excluded if they met any of the following criteria: (1) severe hemodynamic instability, evidenced by increased dose of vasoactive drugs or a mean arterial pressure ≤ 65 mmHg within the preceding two hours, (2) severe cardiac impairment, characterized by a left ventricular ejection fraction < 20% accompanied by episodes of life-threatening arrhythmias or signs of myocardial ischemia in the past 24 hours, (3) a history of pneumothorax or untreated tension pneumothorax within the last six months, (4) a history of hemoptysis requiring embolization within the past 12 months, (5) pregnancy, (6) contraindications to electrical impedance tomography (EIT), including pacemaker placement, automated implantable cardioverter-defibrillator, thoracic or spinal cord injury, or recent thoracic surgery, and (7) refusal to provide informed consent.

### Randomization and blinding

Following enrollment, patients were randomly assigned in a 1:1 ratio to receive CHFO plus usual care (CHFO group) or usual care (control group) using the opaque sealed envelopes. The trial statistician generated the randomization sequence using a web-based randomization system. Researchers were unblinded to treatment allocation due to the nature of the intervention. However, the statistical analyses were conducted in a blinded manner.

### Trial interventions

After enrollment, patients were ventilated with a ventilator (SV800; Mindray, Shenzhen, China) in a volume-controlled mode for a 30-minute stabilization period. Tidal volumes were targeted to maintain driving pressure (DP) below 15 cmH_2_O and plateau pressure (Pplat) at or below 30 cmH₂O. The fraction of inspired oxygen (FiO_2_) was set to achieve an oxygen saturation (SpO_2_) between 90% and 98%. Respiratory rates (RR) was adjusted to keep the arterial carbon dioxide partial pressure (PaCO₂) between 35 and 45 mmHg. Positive end-expiratory pressure (PEEP) was adjusted based on the lower PEEP/FiO_2_ table. The levels of sedation and analgesia were assessed using the Richmond Agitation-Sedation Scale (RASS) and the Critical Care Pain Observation Tool (CPOT), with RASS scores maintained between -2 and 0 and CPOT scores between 0 and 2.

Patients in both groups were positioned supine, with the head of bed elevated to 30 ° to 45 °. After randomization, patients in the control group received secretion aspiration only, whereas those in the CHFO group received CHFO plus usual care. CHFO preparation involved the use of the MetaNeb® system, a T-tube, and a black ring (Model PMN4; Baxter, Singapore) (Figure S1). During the intervention period, the ventilatory mode was switched to pressure-controlled ventilation, with the pressure trigger set to -20 cmH₂O, the high-pressure alarm set to 40 cmH₂O, and the alarms for tidal volume and minute ventilation increased. The alarm of RR was set to 40 breaths per minute. Initially, the low-frequency oscillation mode (170 oscillations per minute) was used and then, based on the patient’s tolerance, switched to the high-frequency oscillation mode (230 oscillations per minute) for a duration of 10 minutes. The termination criteria for CHFO are detailed in the supplementary information. Following CHFO, patients received secretion aspiration, which was performed on an as-needed basis using a closed suction system, with suction applied for no longer than 15 second per attempt. The detailed aspiration procedures are provided in the supplemental information.

During the study period, apart from the CHFO and secretion aspiration performed as part of the intervention protocol, no additional clinical interventions that could affect respiratory status were allowed. Changes in patient positioning, additional airway suctioning beyond routine care, and any adjustments to ventilator mode or ventilator parameters (including tidal volume, PEEP, RR, and FiO₂) were prohibited.

### Data collection

The following variables were collected at enrollment: age, gender, height, weight, body mass index (BMI), primary diagnosis, Acute Physiology and Chronic Health Evaluation II (APACHE II) score, Sequential Organ Failure Assessment (SOFA) score, duration of mechanical ventilation prior to enrollment, and 24 -h sputum volume prior to enrollment.

Vital signs, cough peak flow (CPF), blood gas analysis (PaCO_2_, PaO_2_/FiO_2_), respiratory mechanics (DP, Raw, and Crs), lung morphology assessed using CT scan, and EIT-based data were recorded 30 minutes after previous aspiration (baseline), three minutes after CHFO and suctioning (T0), one hour post-intervention (T1), and three hour post-intervention (T3). EIT data were acquired using a 16-electrode silicone EIT belt positioned at the fourth to fifth intercostal spaces and connected to a commercial device (PulmoVista 500, Dräger Medical, Lübeck, Germany). Data were sampled at 50 Hz with standard low-pass filtering and stored for offline analysis. Detailed procedures for EIT measurements and data analysis are presented in the supplemental information. EIT-based variable, including the percentage of dorsal lung ventilation, global inhomogeneity (GI), and regional ventilation delay (RVD), were calculated for each session [24].

All patients received a remifentanil loading dose to transiently suppress spontaneous breathing prior to respiratory mechanics measurements, with body position kept unchanged. A more detailed description of the study methodology can be found in the supplementary information (Figure S2).

### Study outcomes

The primary outcome was the change in Raw (ΔRaw: R_post-intervention_ - R_baseline_), defined as the difference between the baseline value and the values at time points T0, T1, and T3. Secondary outcomes included changes in Crs, PaO_2_, PaCO_2_, PaO_2_/FiO_2_, the percentage of dorsal lung ventilation, GI index, and RVD index.

### Statistical analysis

Based on our pilot data, the mean decrease in Raw at T1 was 2.86 cmH₂O/L·s with a standard deviation (SD) of 1.33 in the control group, while the mean decrease in Raw was 4.16 cmH₂O/L·s, with an SD of 1.69 in the CHFO group. Assuming a Type I error rate (α) of 0.05 (two-tailed) and a power of 0.8, 23 patients were needed in each group.

Continuous variables were reported as mean ± SD for normally distributed data or median (interquartile range, IQR) for non-normally distributed data. Categorical variables were reported as number (percentage). Group comparisons for categorical data were performed using the chi-square test or Fisher’s exact test, as appropriate. For continuous variables, the Wilcoxon rank-sum test was used. Repeated-measures ANOVA with Mauchly’s test of sphericity (and Greenhouse-Geisser correction if p < 0.05) was used to assess the effects of time, group, and their interaction on the measured variables. When significant effects were detected, post hoc pairwise comparisons were performed with Bonferroni correction.

Exploratory subgroup analyses were performed for ΔRaw at T3 within the following groups (variables measured before randomization): CT phenotype (focal or non-focal); primary diagnosis (pneumonia or non-pneumonia); respiratory system compliance (≤46.5 or ≥46.5 mL/cmH_2_O); and CPF (≤70.8 or >70.8 L/s). To characterize factors associated with therapeutic efficacy, a post hoc analysis was performed wherein patients were stratified into “high-responder” and “low-responder” subgroups based on the median value of ΔRaw at T3. Baseline characteristics and physiological parameters were compared between these two subgroups.

A p value less than 0.05 (two-sided) was considered as statistically significant. Statistical analyses were conducted using SPSS version 27.0 (SPSS Inc., Chicago, IL, USA) and GraphPad Prism 9.4.1.

## Results

### Patient characteristics

Between April 2023 and March 2024, a total of 55 patients were screened for inclusion. Of these, 23 patients per group were finally included in the analysis (Figure S3). All patients tolerated CHFO well. Patient characteristics at enrollment were well balanced between the two groups (Table 1). The mean age was 67.5 ± 13.5 years, 30 of 46 (65.2%) patients were male, and the mean BMI was 23.6 ± 4.4 kg/m^2^. The median 24 -h sputum volume before enrollment was 160.0 [160.0, 180.0] mL. The mean baseline Raw was 13.3 ± 3.4 cmH_2_O/L/s and the median baseline CPF was 67.0 [38.0, 106.5] L/s.

**Table 1:** Baseline characteristics between the two groups.

	All (n = 46)	Control group (n = 23)	CHFO group (n = 23)	*P*
Male, n (%)	30 (65.2)	13 (56.5)	17 (73.9)	0.216
Age, yr	67.5 ± 13.5	65.7 ± 12.2	69.3 ± 14.5	0.370
Body mass index, kg/ m^2^	23.6 ± 4.4	24.4 ± 3.7	22.9 ± 4.9	0.258
APACHE II	22.9 ± 6.1	23.7 ± 6.1	22. 1 ± 6.1	0.411
SOFA	9.1 ± 3.1	9.0 ± 3.4	9.2 ± 2.7	0.852
Sputum volume, ml	165 [160, 180]	160 [155, 180]	170 [160, 185]	0.578
Duration of MV before randomization, d	9.0 [4.0, 13.0]	10.0 [4.0, 13.0]	8.0 [4.0, 12.0]	0.567
Smoking history, n (%)	19 (41.3)	8 (34.8)	11 (47.8)	0.369
Primary diagnosis				
Pneumonia, n (%)	14 (30.4)	7 (30.4)	7 (30.4)	1.000
Intestinal perforation, n (%)	2 (4.3)	1 (4.3)	1 (4.3)	1.000
Neurologic disease, n (%)	19 (41.3)	8 (34.8)	11 (47.8)	0.369
Paraplegia, n (%)	4 (8.7)	3 (13.0)	1 (4.3)	0.295
Multiple rib fractures, n (%)	3 (6.5)	1 (4.3)	2 (8.7)	0.550
Others, n (%)	4 (8.7)	3 (13.0)	1 (4.3)	0.295
Ventilator settings				
Tidal volume, ml/kg PBW	6.9 [6.5, 7.7]	7.1 [6.5, 7.7]	6.8 [6.4, 7.7]	0.328
PEEP, cmH_2_O	6.0 [5.0, 8.0]	6.0 [5.0, 8.0]	6.0 [5.0, 7.0]	0.648
FiO_2_	0.35 [0.30, 0.40]	0.35 [0.30, 0.40]	0.35 [0.35, 0.40]	1.000
Respiratory mechanics				
Driving pressure, cmH_2_O	9.4 [8.5, 10.8]	9.5 [8.7, 11.3]	9.2 [8.1, 10.0]	0.184
Crs, mL/cmH_2_O	46.2 ± 12.7	44.1 ± 13.2	48.8 ± 12.8	0.179
Raw, cmH_2_O/L·s	13.3 ± 3.4	13.5 ± 3.8	13. 1 ± 3.0	0.674
Gas exchange				
pH	7.43 ± 0.04	7.43 ± 0.04	7.42 ± 0.03	0.295
PaCO_2_, mmHg	36.7 ± 5.8	36.6 ± 5.3	37.1 ± 6.6	0.662
PaO_2_/FiO_2_, mmHg	267.5 ± 90.9	263.4 ± 93.8	269.2 ± 81.9	0.829
EIT-based data				
Dorsal lung ventilation, %	36.6 ± 9.8	36. 1 ± 13.1	36.8 ± 8.8	0.835
GI index	0.377 ± 0.057	0.389 ± 0.072	0.365 ± 0.034	0.135
RVD index	3.76 ± 1.46	3.61 ± 1.62	3.91 ± 1.28	0.473
Focal lung lesion on imaging*	29 (63.0)	10 (43.5)	19 (82.6)	0.013
CPF, L/s	61.6 [37.9, 100.6]	67.0 [37.4, 106.5]	53.6 [38.0, 98.6]	0.089

Values are represented as count (percentage), mean ± SD, or median (interquartile range).

*CHFO* Continuous high-frequency oscillation, *APACHE II* Acute Physiology and Chronic Health Disease Classification System II, *SOFA* Sequential Organ Failure Assessment, *MV* mechanical ventilation, *PBW* predicted body weight, *PEEP* positive end-expiratory pressure, *FiO_2_* fraction of inspired oxygen, *Crs* respiratory system compliance, *PaCO_2_* arterial partial pressure of blood carbon dioxide, *PaO_2_/FiO_2_* the ratio of partial pressure arterial oxygen and fraction of inspired oxygen, *EIT* Electrical impedance tomography, *GI* Global inhomogeneity, *RVD* regional ventilation delay, *CPF* cough peak flow.

* Lung morphology was assessed before randomisation using a CT scan of the whole lung or chest x-ray. The focal lung lesion was defined as presence of consolidations localised only in the lower and back part of the lungs.

### Effect of CHFO on the airway resistance

**Fig. 1:**
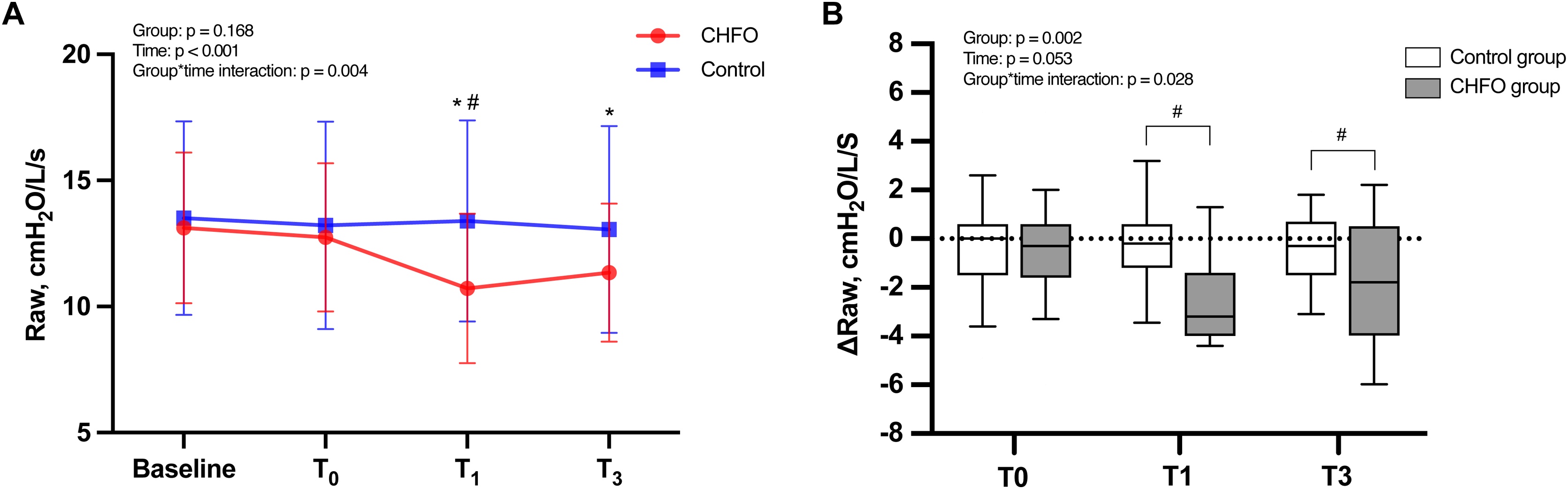
Effect of CHFO on the airway resistance. The figure shows Raw (A), ΔRaw (the change in Raw: RT - Rbaseline) (B) at at 30 minutes after previous aspiration (baseline), three-minute post-intervention (T0), one-hour post-intervention (T1), and three-hour post-intervention (T3) in both groups. Data in the graphs A are presented as the median (filled symbols) with 95% CI (whiskers). Graph B presents the data using box plots, The bottom and top of the box indicate the 25th and 75th percentile, the horizontal band near the middle of the box is the median, and the ends of the whiskers represent the 10th and 90th percentiles. Data in the graphs A are presented as the median (filled symbols) with 95% CI (whiskers). Statistically significant p values within study arms are reported in the figures, *p < 0.05 for data compared with baseline in the CHFO group. ^#^p < 0.05 for data CHFO vs. control group in the same timepoint. *CHFO* continuous high-frequency oscillation, *Raw* airway resistance.

For Raw, CHFO had a lower Raw at T1 in the CHFO group (10.7 ± 3.0 cmH_2_O/L/s) compared to control group (13.4 ± 4.0 cmH_2_O/L/s, p < 0.05). There were no differences between the two groups at baseline, T0, or T3 (Table S1). Compared to baseline, Raw in the CHFO group was significantly lower at T1 and T3 (both p < 0.05), whereas there was no difference between baseline and T0. In the control group, there was no difference between Raw at baseline, T0, T1, and T3 (Fig. 1A). The individual trajectories of Raw over time in the both groups were presented in the Figure S4.

ΔRaw at T0 did not differ between the two groups. ΔRaw at T1 was significantly higher in the CHFO group (-2.4 ± 1.8 cmH_2_O/L/s) compared to control group (-0.1 ± 1.6 cmH_2_O/L/s, p < 0.001). Similarly, there was a higher ΔRaw at T3 in the CHFO group (-1.8 ± 2.4 cmH_2_O/L/s) compared with control group (-0.5 ± 1.9 cmH_2_O/L/s, p < 0.05) (Fig. 1B).

### Effect of CHFO on the respiratory mechanics

For ΔCrs, there was no difference between the two groups at T0. ΔCrs was higher in the CHFO group than control group at T1 and T3 (both p < 0.05) (Table 2). Similarly, Crs was higher in the CHFO group than control group at T1 and T3 (both p < 0.05). In the CHFO group, Crs significantly increased at T1 and T3 compared to baseline (both p < 0.05), whereas no change was observed in the control group. No significant differences in the Pplat or DP were observed at T0, T1, and T3 between groups (Table S1, Fig. 2).

**Table 2:** Secondary outcomes between two groups.

	Control group (n = 23)			CHFO group (n = 23)		
	T0	T1	T3	T0	T1	T3
Respiratory mechanics						
ΔCrs, mL/cmH_2_O	44.1 ± 13.2	−0.3 ± 4.2	−1.0 ± 5.8	2.3 ± 6.4	4.9 ± 8.8*	4.3 ± 9.6*
Gas exchange						
ΔPaCO_2_, mmHg	0.0 ± 3.0	0.2 ± 3.7	1.7 ± 5.4	−0.8 ± 4.3	−0.1 ± 4.4	−0.5 ± 5.0
ΔPaO_2_/FiO_2_, mmHg	16.8 ± 52.8	7.0 ± 51.5	8.4 ± 57.2	37.7 ± 70.1	4.4 ± 48.0#	24.8 ± 55.6
EIT-based data						
ΔDorsal lung ventilation, %	1.0 (-3.0, 3.0)	1.0 (-3.0, 3.0)	1.0 (-1.0, 6.0)	−2.0 (-4.0, 1.0)	4.0 (2.0, 8.0)*	5.0 (-1.0, 9.0)
ΔGI index	0.00 ± 0.03	−0.00 ± 0.03	−0.00 ± 0.04	−0.01 ± 0.03	−0.00 ± 0.03	−0.01 ± 0.03
ΔRVD index	0.13 ± 1.21	−0.04 ± 1.45	−0.30 ± 2.13	−0.13 ± 1.37	−0.52 ± 1.41	0.13 ± 1.83

Values are represented as count (percentage), mean ± SD, or median (interquartile range).

*ΔCrs* the change in respiratory system compliance (R_post-intervention_ - R_baseline_), *ΔRaw* the change in Raw (R_post-intervention_ - R_baseline_), *ΔPaCO_2_* the change in arterial partial pressure of blood carbon dioxide (R_post-intervention_ - R_baseline_), *ΔPaO_2_/FiO_2_* the change in the ratio of partial pressure arterial oxygen and fraction of inspired oxygen (R_post-intervention_ - R_baseline_), *ΔDorsal lung ventilation* the change in dorsal lung ventilation (R_post-intervention_ - R_baseline_). ^a^p < 0.05, CHFO vs. control at the same timepoint.

*CHFO* Continuous high-frequency oscillation, *Raw* Airway resistance, *Crs* respiratory system compliance, *PaCO_2_* arterial partial pressure of blood carbon dioxide, *PaO_2_/FiO_2_* the ratio of partial pressure arterial oxygen and fraction of inspired oxygen, *EIT* Electrical impedance tomography, *GI* Global inhomogeneity, *RVD* regional ventilation delay.

***p < 0.001 for data CHFO vs. control group in the same timepoint.

* p < 0.05 for data CHFO vs. control group in the same timepoint.

# p < 0.05 for data compared with T0 in the same group.

**Fig. 2:**
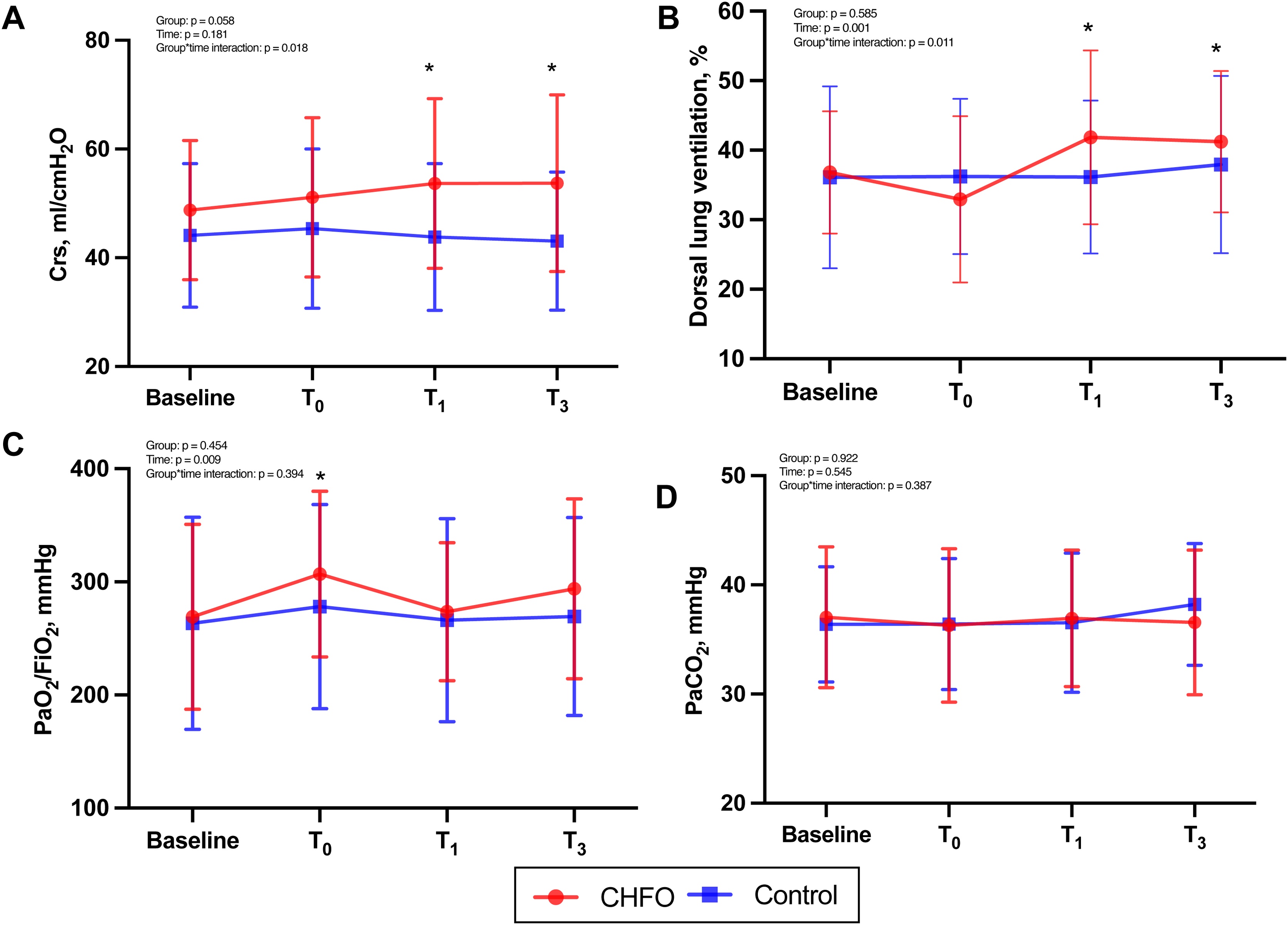
Effect of CHFO on the secondary outcomes. The figure shows Crs (A), dorsal lung ventilation (B), PaO_2_/FiO_2_ (C), and PaCO_2_ (D) at 30 minutes after previous aspiration (baseline), three-minute post-intervention (T0), one-hour post-intervention (T1), and three-hour post-intervention (T3) in both groups. Data are presented as the median (filled symbols) with 95% CI (whiskers). *p < 0.05 for data compared with baseline in the same group (CHFO group). *CHFO* continuous high-frequency oscillation, *Crs* respiratory system compliance, *PaO_2_/FiO_2_* the ratio of partial pressure arterial oxygen and fraction of inspired oxygen, *PaCO_2_* arterial partial pressure of blood carbon dioxide.

### Effect of CHFO on gas exchange and ventilation distribution

There were no differences in ΔPaO_2_/FiO_2_ and ΔPaCO_2_, between the two groups at T0, T1, or T3 (Table 2). PaO_2_/FiO_2_ increased significantly at T0 compared to baseline (p < 0.05), and no significant differences were observed at T1 or T3 compared to baseline. No significant differences in PaCO₂ were observed at T0, T1, or T3 compared to baseline (Fig. 2).

Δdorsal lung ventilation was higher in the CHFO group than control group at T1 (CHFO: 4.0 [2.0, 8.0] vs control: 1.0 [-3.0, 3.0]%, p < 0.05), whereas no differences were found in both groups at T0 and T3 (Table 2). In the CHFO group, dorsal lung ventilation at T1 and T3 was significantly higher than baseline, whereas it was slightly lower at T0 compared to baseline, although this decrease did not reach statistical significance (Table S1, Fig. 2). ΔGI and ΔRVD index did not differ in the two groups at different timepoints.

**Fig. 3:**
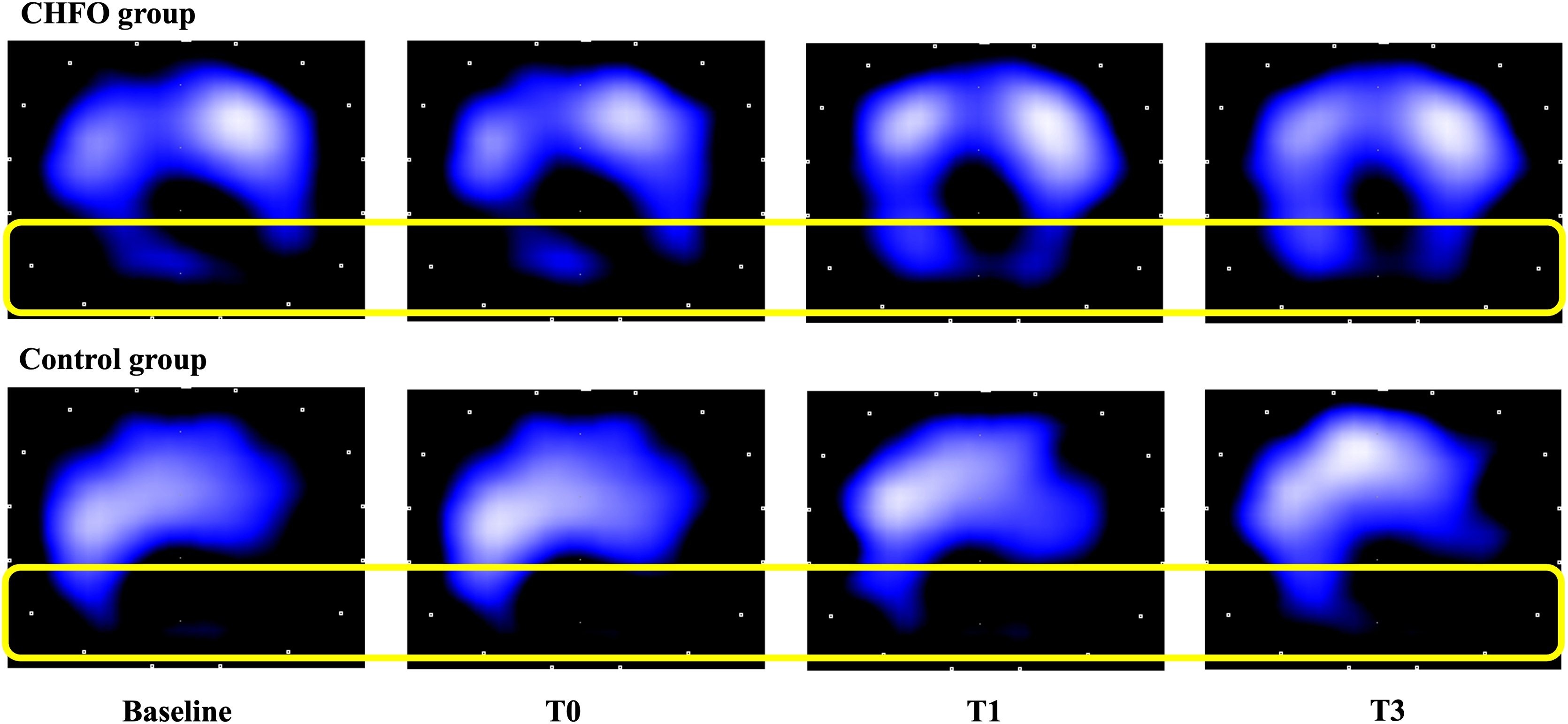
The physiological effect of CHFO treatment and usual care on the EIT-based ventilation distribution. The figure shows ventilation distribution at 30 minutes after previous aspiration (baseline), three-minute post-intervention (T0), one-hour post-intervention (T1), and three-hour post-intervention (T3) in both groups. *CHFO* continuous high-frequency oscillation, *EIT electrical impedance tomography.*

The physiological effects of CHFO and usual care on EIT-based ventilation distribution in two representative patients are presented in the Fig. 3.

### Subgroup analysis

ΔRaw at T3 was significantly lower in the CHFO group than in the control group among patients with lower baseline Crs and in those with higher baseline CPF. No differences in ΔRaw at T3 between the groups were observed in the other patient subgroups (Figure S5).

### High-responders versus low-responders

Based on the median ΔRaw at T3, 21 patients with ΔRaw > −1.1 cmH₂O/L/s were classified as high responders and 25 patients with ΔRaw ≤ −1.1 cmH₂O/L/s as low responders. Compared with low responders, high responders had a lower percentage of dorsal lung ventilation and more frequent focal lung lesions (Table S2).

## Discussion

In this randomized trial involving mechanically ventilated patients with excessive airway secretions, we found that CHFO group showed a significantly larger decrease in Raw and increase in Crs compared to control group after one- and three-hour post-intervention. Additionally, increase in dorsal lung ventilation from baseline was greater in the CHFO group compared to control group after one-hour post-intervention.

## Effect of CHFO on the airway resistance

CHFO reduced Raw in mechanically ventilated patients in our trial. The accumulation of airway secretions, frequently aggravated by comorbidities, can impair mucociliary clearance and increase airway resistance [4,15]. CHFO facilitates secretion clearance by generating intratracheal pressure oscillations that disrupt mucus cohesion, mobilizing secretions from the distal to the proximal airways [21,25]. This mechanism helps clear proximal airway secretions through cough, improving bronchial structure and patency, and reducing Raw. Our findings suggest that peak airway pressure reduction reported by Morgan et al. [15] may be mediated by the decreased Raw.

Importantly, patients with lower Crs and higher CPF appeared to derive greater benefit from CHFO therapy, suggesting a phenotype characterized by increased airway obstruction and secretion burden rather than diffuse lung involvement. These physiological features are commonly observed in conditions such as pneumonia-predominant disease, secretion-related atelectasis, or dependent consolidation, where mucus plugging and heterogeneous airway narrowing play important roles in respiratory mechanics. These findings support the concept that baseline airway mechanics may help identify target populations most likely to benefit from CHFO.

Furthermore, our results showed that patients with a greater therapeutic response exhibited a lower percentage of dorsal lung ventilation and more focal lung involvement. Such regional imaging patterns may provide complementary information to physiological measurements, enabling a more refined identification of patients with mechanically reversible airway obstruction. Beyond CHFO, this phenotype-oriented approach may have broader implications for guiding airway clearance strategies and other individualized respiratory interventions, highlighting the potential role of combined physiological and imaging assessments in precision respiratory care.

This study also demonstrated that the effect of CHFO in reducing Raw persists for three hours post-intervention [[26], [27], [28], [29]]. The increase in Raw at T3 compared to T1 suggests possible sputum re-accumulation in distal airways, reflecting a time-dependent decline in airway clearance efficacy. Based on these findings, it is hypothesized that an optimal treatment frequency for CHFO may be approximately once every hour. Further research is needed to validate this hypothesis, as excessively frequent treatments could present additional clinical challenges.

## Effect of CHFO on the respiratory system compliance and ventilation distribution

Airway secretion retention may induce multiple pathophysiological consequences, including (1) luminal obstruction, potentially causing lung tissue atrophy or collapse, (2) reduced pulmonary compliance, and (3) preferential accumulation of secretions in the dorsal regions, impairing regional ventilation in the supine position. By delivering high-frequency oscillations, CHFO can reduce mucus retention and support alveolar recruitment, thereby improving ventilation distribution. The present study supports previous findings that CHFO improves Crs in mechanically ventilated patients, consistent with earlier research demonstrating an increased percentage of dorsal lung ventilation in the CHFO group as measured by EIT [7,30]. Notably, we observed an immediate post-CHFO reduction in dorsal lung ventilation, followed by recovery and subsequent increases at later timepoints. This transient redistribution suggests that treatment may initially favor gas delivery to already well-ventilated ventral regions. As pressures stabilize, a re-equilibration of gas distribution occurs, potentially facilitating the recruitment of collapsed or poorly ventilated dorsal areas. These dynamic shifts, although short-lived, underscore the importance of monitoring regional ventilation time-courses [[31], [32], [33]] to fully understand the physiologic impact of CHFO. Further studies are needed to clarify the clinical implications of these regional ventilation changes.

## Effect of CHFO on gas exchange

No significant changes were observed in the PaO_2_/FiO_2_ and PaCO_2_ after CHFO in this trial. The findings are in contrast with those reported in prior studies, which generally showed that CHFO improved gas exchange [34]. Previous studies have primarily assessed the long-term effects of CHFO [21], while large physiological studies evaluating its acute impact on gas exchange remain notably scarce. Several reasons may explain these results. First, this study evaluated acute physiological effects of CHFO. Second, enrolled patients had normal baseline PaCO_2_, and mechanical ventilation settings (mode/parameters) were strictly standardized throughout the protocol. Under these controlled conditions-with neither pre-existing hypercapnia nor adjustments to ventilatory support-CHFO demonstrated no significant impact on oxygenation or PaCO_2_. Further studies are warranted to determine whether CHFO has a beneficial effect on gas exchange in mechanically ventilated patients with hypercapnic respiratory failure.

## Limitations

This study has several limitations. First, the interpretation of respiratory mechanics may be confounded by the concurrent standard care procedures. Specifically, airway suctioning inherently clears secretions, contributing to an immediate reduction in Raw, while the deep sedation required for accurate measurement can alter chest wall tone, potentially influencing Crs and the temporal pattern of physiological changes. However, it is important to note that our study used a randomized controlled design where the control group received the identical sedation and suctioning-only protocol. Therefore, the confounding effects of these background procedures were balanced between groups, suggesting that the superior and sustained improvements in Raw observed in the CHFO group reflect the specific therapeutic efficacy of CHFO. Second, as a short-term physiological evaluation limited to a three-hour observation period, this trial cannot assess the potential sustained effects of CHFO beyond this timeframe, such as 24 -h or longer-term outcomes. Third, while standardized sputum aspiration protocols were implemented, procedural consistency was inherently limited by inter-operator variability, particularly in patients with impaired cough reflexes. This variability potentially delayed secretion clearance even in cases of confirmed proximal airway mucus accumulation. Furthermore, quantitative sputum volume measurements were confounded by daily hydration status and humidification variables [35,36]. Finally, respiratory measurements may have been affected by spontaneous breathing because patients were maintained at a RASS score between -2 and 0. Under these conditions, respiratory muscle activity may lead to underestimation and increased variability of Raw, because standard resistance calculations assume fully passive breathing. These potential sources of bias indicate that our results should be interpreted with caution.

## Conclusions

In mechanically ventilated patients with excessive airway secretions, CHFO group had a greater decrease in the Raw and increase in the Crs compared to control group after one- and three-hour post-intervention. Moreover, CHFO group had a higher increase in the dorsal lung ventilation compared to control group after one-hour post-intervention in these patients. These benefits were maintained throughout the three-hour post-intervention period.

## Author contributions

Yuxuan Wang and Dongyu Chen collected the data, interpreted the results and drafted the manuscript. Rui zhang, Haiying Wu and Xinxin Guo accessed and verified the underlying data. Ling Liu designed the study and performed the statistical analysis. Ling Liu, Xueyan Yuan and Wei Xie interpreted the results, and revised the manuscript for important intellectual content. All authors had full access to all the data in the study and had final responsibility for the decision to submit for publication.

## Funding

This work was supported by National Science and Technology Major Project (2024ZD0530000), the National Natural Science Foundation of China (U25C2018, 82270083, 82470079,82270083), Jiangsu Province Science and Technology Plan Project 'Provincial Frontier Technology R&D Program' (BF2024054), Jiangsu Provincial Medical Key Laboratory (ZDXYS202205), Yunnan Province Science and Technology commission (202305AF150186).

## Ethics approval and consent to participate

The study was conducted in accordance with the CONSORT guideline for the report of randomized controlled trial. The study protocol was reviewed and approved by the Human Ethics Committee of the Institutional Review Board of Zhongda Hospital (reference number 2023ZDSYLL108-P01).

## Competing interest

The authors declare no competing interest in relation with the work.

## Consent for publication

All authors approved the final version to be published and agree to be accountable for all aspects of the work.

## Data availability

The datasets used and/or analysed during this study are not publicly available but are available from the corresponding authors on reasonable request.
